# Increased MicroRNA-630 Expression in Gastric Cancer Is Associated with Poor Overall Survival

**DOI:** 10.1371/journal.pone.0090526

**Published:** 2014-03-12

**Authors:** Dake Chu, Zhengwei Zhao, Yunming Li, Jipeng Li, Jianyong Zheng, Weizhong Wang, Qingchuan Zhao, Gang Ji

**Affiliations:** 1 State Key Laboratory of Cancer Biology and Xijing Hospital of Digestive Diseases, Xijing Hospital, Fourth Military Medical University, Xi'an, Shaanxi, China; 2 State Key Laboratory of Cancer Biology and Department of Biochemistry and Molecular Biology, Fourth Military Medical University, Xi'an, Shaanxi, China; 3 Department of General Surgery, Tangdu Hospital, Fourth Military Medical University, Xi'an, China; 4 Department of Medical Affair, General Hospital of Chengdu Military Region, Chengdu, Sichuan, China; University of Barcelona, Spain

## Abstract

MicroRNAs are noncoding RNAs that regulate multiple cellular processes during cancer progression. Among various microRNAs, MiR-630 has recently been identified to be implicated in many critical processes in human malignancies. We aimed to investigate the significance and prognostic value of miR-630 in human gastric cancer. Gastric cancer and adjacent normal specimens from 236 patients from who had not received neoadjuvant chemotherapy were collected. The expression of miR-630 was investigated by quantitative real-time PCR assay and its association with overall survival of patients was analyzed by statistical analysis. MiR-630 expression level was significantly elevated in gastric cancer in comparison to adjacent normal specimens. It is also proved that miR-630 expression was to be associated with gastric cancer invasion, lymph node metastasis, distant metastasis and TNM stage. In addition, survival analysis proved that elevated miR-630 expression was associated with poor overall survival of patients. Multivariate survival analysis also proved that miR-630 was an independent prognostic marker after adjusted for known prognostic factors. The present study proved the over-expression of miR-630 and its association with tumor progression in human gastric cancer. It also provided the first evidence that miR-630 expression was an independent prognostic factor for patients with gastric cancer, which might be a potential valuable biomarker for gastric cancer.

## Introduction

Gastric cancer is one of the most common malignant diseases worldwide [1]. The prognosis of gastric cancer is poor and patients are generally diagnosed at a rather advanced stage in most countries [2], [3]. Thus, the overall 5-year survival rate is about 40%, and that of the patients with distant metastasis is less than 5% [4]–[8]. Approximately 700,000 gastric cancer patients die annually, making it the second most common cause of cancer-related death worldwide. Data showed that the crude mortality rate of gastric cancer was 25.2 per 100,000 (32.8 per 100,000 in men and 17.0 per 100,000 in women) [1]. But there exists remarkable geographical variation in incidence of gastric cancer for eastern countries such as China and Japan have highest rates of gastric cancer than western countries [9], [10]. In China, gastric cancer is the second most frequently diagnosed cancer and the third leading cause of cancer death, with an estimated 464 439 new cases and 352 315 cancer deaths in 2008. The overall estimated age-adjusted incidence rate in 2008 was 29.9 per 100,000 people in China [9]. So, gastric cancer remains a significant problem worldwide despite a declining incidence in not only western countries but also China.

MicroRNAs (miRNAs) are a class of highly conserved, single-stranded, small noncoding RNA molecules, which are known as endogenous regulators of post-transcriptional gene expression regulating expression through translational repression and messenger RNA cleavage [11]. They can inhibit gene expression at the posttranscriptional level by binding to the 3′ untranslated region of target mRNAs can result in mRNA degradation or translation inhibition, depending on the degree of complementary base pairing [12], [13]. It has been widely accepted that miRNAs play pivotal roles in various biological processes, including development, cell proliferation, differentiation, apoptosis and metabolism [14]. Accumulated evidence also suggests that miRNAs participate in the tumor angiogenesis, invasion and metastasis of human malignancy [15]. Aberrant miRNA expression has been associated with oncogenesis, and some miRNAs act as tumor suppressors, whereas others as oncogenes, depending on their targets, which may provide insight into the diagnosis and prognosis of human malignancies [16]. Recently, evidences have shown that expression of miRNAs is deregulated in various types of human malignancies such as gastric cancer, breast cancer, colorectal cancer, prostate cancer, lung cancer, pancreas cancer and chronic lymphocytic leukemia [17]–[19]. In human gastric cancer, multiple miRNAs with aberrant expression have been identified, for example, miR-21, miR-22, miR-23, miR-127, miR-148, miR-202 and miR-433, which played oncogenic or suppressive role [20]–[25]. These results indicated that miRNAs can play diverse and crucial roles in human malignancies including gastric cancer. However, the expression profile of miRNAs is highly tissue and cancer type specific and miRNA specific, thus demonstrating the functional and clinical significance of a specific miRNA may provide clinically relevant insights into miRNA function and efficacious cancer management [12], [15]. To our knowledge, till now, the clinical and prognostic significance of miR-630 expression in gastric cancer has not been reported yet.

In the present study, we have investigated the expression level of miR-630 in clinical gastric specimens and adjacent normal tissues, as well as analyzing its association with overall survival of patients.

## Materials and Methods

### Patients and Specimens

The present study has been approved by the Ethics Committee of the Fourth Military Medical University. All members involved have provided written informed consent. Fresh clinical gastric cancer specimens and adjacent normal tissues were collected from 236 patients who underwent surgery between January 2007 and December 2008 in Xijing Hospital of Digestive Diseases, Fourth Military Medical University. None of these patients had received chemotherapy prior to surgery. In addition, normal tissue samples were taken from 18 patients who underwent surgery for reasons other than malignancy as normal control samples. The histomorphology of all tissue specimens were confirmed by the Department of Pathology, Fourth Military Medical University. Specimens were put immediately into liquid N_2_ after surgical resection for 10 min, then into a −70°C ultra-freezer for mRNA isolation. Patients' clinical information, such as age, sex, differentiation status and TNM stage, was collected and stored in a database. Complete follow-up was made available for at least 5 years. Overall survival is defined as the time elapsed from surgery to death of patients with gastric cancer. Follow-up information of all participants was updated every three month by telephone visit and questionnaire letters. Death of participants was ascertained by reporting from the family and verified by review of public records.

### Quantitative Real-time PCR

Total RNA was purified from all the 236 gastric cancer and matching adjacent normal specimens by the manufacturer using Trizol reagent (Invitrogen, Carlsbad, CA, USA). Only those total RNA samples with OD A260/A280 ratio close to value of 2.0, which indicates that the RNA is pure, were subsequently analyzed. The miR-630 and RNU44 internal control specific cDNA were synthesized from total RNA using gene-specific primers according to the TaqMan MicroRNA assays protocol (Applied Biosystems, Foster City, CA, USA). The reverse transcription products were then amplified and detected by real-time PCR using Taqman MicroRNA Assay (Applied Biosystems) specific for hsa-miR-630. Each sample was examined in triplicate and the raw data were presented as the relative quantification of miR-630 expression evaluated by the comparative cycle threshold (CT) method, normalized with respect to RNU44. Mean normalized miR-630 expression ± standard deviation (SD) was calculated from triplicate analysis. Real-time PCR was performed using an ABI 7500 system (Applied Biosystems) and comparative 2^−ΔΔCt^ analysis was performed using SDS 2.2.2 software (Applied Biosystems).

### Statistical Analysis

Associations between miR-630 expression and clinicopathological characteristics were analyzed by Mann-Whitney test and Kruskal-Wallis test, as appropriate. Survival curves were estimated using the Kaplan-Meier method and differences in survival distributions were evaluated by the log-rank test. Cox's proportional hazards modeling of factors potentially related to survival was performed in order to identify which factors might have a significant influence on survival, and controlling for age, gender and differentiation status. Differences with a *P* value of 0.05 or less were considered to be statistically significant.

## Results

### MiR-630 Expression Detected in Gastric Cancer

In the real-time PCR assay, 236 cases of gastric cancer, matching adjacent normal specimens and 18 cases of normal control specimens were investigated. Relative expression of miR-630 normalized to RNU44 in gastric cancer was found to be 8.51±1.63 (Mean±SD), while that in matching adjacent normal was 3.25±0.63. Statistical results proved that the expression of miR-630 in gastric cancer was significantly higher than that in adjacent and normal control specimens (*P*<0.05). These results indicated that miR-630 might play an oncogenic role in gastric cancer. To facilitate further analysis on the association between miR-630 expression and clinical features, we manually defined gastric cancer with miR-630 expression lower than 3.88 (Mean+SD expression of miR-630 in adjacent specimens) as low miR-630 expression group while specimens with miR-630 expression no lower than 3.88 were defined as high miR-630 expression group. Thus, among 236 cases of gastric cancer, 98 cases were assigned to low expression group while 138 cases were assigned to high expression group.

### Relationship of MiR-630 to Clinicopathological Characteristics of Gastric Cancer

As our investigation proved that miR-630 expression was increased in gastric cancer, which indicated that miR-630 might act as an oncogenic role in gastric cancer. We further investigated the association of miR-630 expression with clinicopathological characteristics of patients to explore its potential role in gastric cancer progression. Statistical analysis results, showed in Table 1, proved that increased miR-630 expression was associated with advanced gastric cancer invasion since high miR-630 expression was more frequently to be detected in T3 and T4 tumors (*P* = 0.007). As far as tumor metastasis was considered, elevated miR-630 expression was related to positive lymph node and distant metastasis for high miR-630 expression was more frequently to be detected in tumors with positive lymph node (*P*<0.001) or distant metastasis (*P* = 0.002). In addition, when we measured patients' clinical features by TNM staging system, we found that miR-630 expression was significantly associated with TNM stage of gastric cancer because high miR-630 expression was more likely to be detected in tumors with advanced TNM stage (*P*<0.001). These results suggested that miR-630 might play an oncogenic role in the progression of gastric cancer. However, the positive ration of miR-630 was not found to be associated with patients' gender (*P* = 0.788), age (*P* = 0.471), differentiation status (*P* = 0.890), tumor site (*P* = 0.835) or lauren classification (*P* = 0.160).

**Table 1:** Association of miR-630 with clinicopathological characteristics.

Variable	n	miR-630 expression		*P*
		Low	High	
**Total**	236	98	138	
**Gender**				0.788*
Male	130	55	75	
Female	106	43	63	
**Age**				0.471*
<60	138	60	78	
≥60	98	38	60	
**Differentiation**				0.890†
Poor	81	34	47	
Moderate	105	42	63	
Well	50	22	28	
**Tumor site**				0.835†
Gastroesophageal junction	45	20	25	
Corpus/Fundus	139	58	81	
Antrum/Pylorus	52	20	32	
**Lauren classification**				0.160*
Intestinal	110	51	59	
Diffuse	126	47	79	
**Invasion**				0.007*
T1+T2	115	58	57	
T3+T4	121	40	81	
**lymph node Metastasis**				<0.001*
Negative	108	65	43	
Positive	128	33	95	
**Distant Metastasis**				0.002*
Negative	208	94	114	
Positive	28	4	24	
**TNM stage**				<0.001†
I	36	28	8	
II	72	37	35	
III	100	29	71	
IV	28	4	24	

*P value when expression levels were compared using Mann Whitney test.

† *P* value when expression levels were compared using Kruskal Wallis test.

### The Relationship of MiR-630 to Overall Survival of Patients' with Gastric Cancer

**Figure 1:**
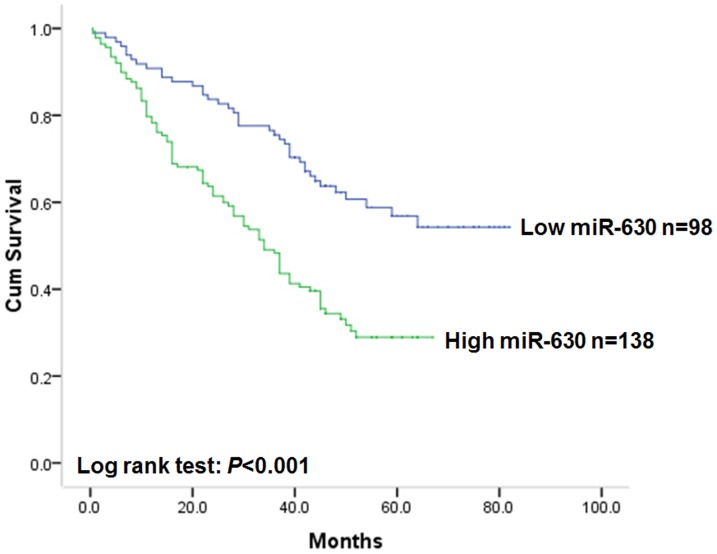
Kaplan-Meier postoperative survival curve for patterns of patients with gastric cancer and miR-630 expression.

During the whole follow-up period, 130 of the 236 patients (55.1%) with gastric cancer had died and the median overall survival time of all recruited patients was 48 months. Kaplan–Meier analysis was applied to examine the prognostic value of miR-630 expression to overall survival of patients with gastric cancer. Results proved that patients with gastric cancer of high miR-630 expression tended to have worse overall survival (log rank test: *P*<0.001, Figure 1). The median survival time of patients with low miR-630 expression can not be estimated because more than 50% of patients survived during the follow-up period. While the postoperative median survival time of patients with high miR-630 expression was 34 months (95% CI: 28.2–39.8). When unadjusted hazard ratio (HR) was considered with low miR-630 expression as reference (1.00), patients with gastric cancer of high miR-630 expression had a 2.28-fold higher risk of death (95% CI: 1.57–3.32; *P*<0.001). As far as clinicopathological characteristics were considered, lymph node metastasis (log rank test: *P* = 0.002), distant metastasis (log rank test: *P*<0.001) and TNM stage (log rank test: *P*<0.001) were also proved to be associated with overall survival since patients with gastric cancer of lymph node metastasis, distant metastasis or advanced grade tended to have worse overall survival and higher risk of death. However, gender (log rank test: *P* = 0.168), age (log rank test: *P* = 0.572) differentiation status (log rank test: *P* = 0.156), tumor site (log rank test: *P* = 0.632) or lauren classification (log rank test: *P* = 0.573) had no prognostic value on overall survival of patients with gastric cancer.

As miR-630 expression was proved to be associated with overall survival of patients in univariate survival analysis, we further investigated whether miR-630 could serve as an independent prognostic marker for patients with gastric cancer. Because lymph node metastasis and distant metastasis information was included in TNM stage data, we performed Cox proportional hazards model, which is adjusted by sex, age and TNM stage. Results showed that the adjusted HR of high miR-630 expression group was 3.51 (95% CI: 1.83–6.72; *P*<0.001). These results proved that miR-630 was an independent prognostic factor of overall survival for patients with gastric cancer. Thus, increased miR-630 expression could be an indicator of poor overall survival without consideration of age, sex or TNM stage (Table 2).

**Table 2:** Association of miR-630 expression and clinical factors with overall survival of patients with gastric cancer.

	Unadjusted HR* (95% CI)	*P*	Adjusted HR† (95% CI)	*P*
**miR-630**				
Low expression	-		-	
High expression	2.28 (1.57–3.32)	<0.001	3.51 (1.83–6.72)	<0.001
**Gender**				
Female	-		-	
Male	1.42 (0.86–2.33)	0.168	1.38 (0.77–2.47)	0.276
**Age**				
<60	-		-	
≥60	1.12 (0.76–1.64)	0.572	1.14 (0.80–1.63)	0.466
**TNM stage**				
I	-		-	
II	1.76 (1.20–2.58)	0.004	1.48 (1.02–2.20)	0.049
III	3.64 (1.84–7.22)	<0.001	3.30 (1.91–5.71)	<0.001
IV	8.82 (4.15–18.96)	<0.001	9.26 (4.37–19.58)	<0.001

*Hazard ratios in univariate models.

† Hazard ratios in multivariable models.

Abbreviations: HR, hazard ratio; 95% CI, 95% confidence interval.

## Discussion

According to recent studies, more than 1,900 human miRNAs have been identified, which are estimated to regulate over 60% of genes in mammals [26]. Due to their great importance in the regulation of gene expression, it has been widely accepted that miRNAs are involved in multiple cellular functions including proliferation, apoptosis and differentiation, thus, have been implemented in diverse physiological and pathological processes ranging from development to cancer [27]–[29]. Previous investigation surveyed expression profiles of miRNA in stage I non-small cell lung cancer to identify patterns that might predict recurrence after surgical resection found that mir-630 was one of the most commonly miRNA among the 1,000 classifiers identified [30]. Another study on non-small cell lung found that miR-630 could regulate cisplatin-induced cancer cell death, indicating miR-630 was a potential modulator of the cisplatin response in non-small cell lung cancer [31]. MiR-630 was also found to be upregulated in head and neck squamous cell carcinoma after cisplatin treatment and can modulate the protein expression of ATG5, ATG6/BECN1, ATG10, ATG12, ATG16L1 and UVRAG [32], [33]. Recent study proved that upregulation of miR-630 in pancreatic cancer cells could induce apoptosis by targeting IGF-1R [34]. Since individual miRNAs in different cancer types can have a large number of different gene targets, thus, have different functions in various malignancies. So, the functional discovery of individual miRNAs may enable deeper insight into regulation of gene expression and complexity of cancer progression.

In the present study, we have investigated miR-630 mRNA expression by real-time PCR assay in 236 cases of gastric cancer from patients who had not received neoadjuvant chemotherapy. Based relative expression calculation, we analyzed the association of miR-630 with clinicopathological characteristics as well as prognosis of patients. Results showed that miR-630 expression was increased in gastric cancer compared with that in adjacent and normal control tissues for high expression of miR-630 was more likely to be detected in gastric cancer specimens, which indicating its possible participation on carcinogenesis. It is also found that miR-630 expression was closely related to gastric cancer invasion, lymph node metastasis, distant metastasis and TNM stage for high expression of miR-630 was more frequently to be detected in tumors with deep invasion, lymph node metastasis, distant metastasis or advanced TNM stage, suggesting the possible participation of miR-630 on gastric cancer invasion and metastasis. Together with the above evidence, it was thus proposed that miR-630 may play important roles in gastric cancer carcinogenesis and progression.

As miR-630 expression was found to be associated with gastric cancer invasion and metastasis, considering the invasion of cancer to nearby tissues and metastasis to distal tissues are crucial factors affecting the prognosis of patients, miR-630 might be a potential prognostic marker for patients with gastric cancer. In order to investigate the prognostic role of miR-630 on gastric cancer, we performed Kaplan-Meier analysis of overall survival. Results showed that patients with gastric cancer of high miR-630 expression tend to have worse overall survival in comparison to patients with tumor of low miR-630 expression, which suggested that miR-630 expression was a prognostic marker for patients with gastric cancer. To further evaluate the prognostic value of miR-630 in gastric cancer, we performed Cox proportional hazards model which was adjusted for gender, age and TNM stage of patients. Results proved that increased miR-630 expression was a marker of poor overall survival independent of adjusted factors, thus, miR-630 could be utilized to determine patients' prognosis with out considering TNM stage. These results indicated that miR-630 could constitute a molecular prognostic marker additive to TNM stage for patients with gastric cancer, identifying high risk individuals who are more likely to have tumor relapse in clinical practice, thus, good candidates to receive more aggressive treatment. These results were in consistent with investigations focused on non-small cell lung cancer, indicating the consistence of miR-630 function in these types of tumor. Thus, the positive linkage between miR-630 overexpression and poor prognosis may not only be used for identifying gastric cancer patients with higher risk of early tumor relapse but also for providing valuable clues to understand the possible mechanism of gastric cancer invasion and metastasis.

In conclusion, we have proved that miR-630 expression was increased in gastric cancer and associated with tumor progression. The present study also demonstrated for the first time that miR-630 expression was an independent prognostic factor of patients with gastric cancer. Therefore, it is possible that miR-630 may play an important role in invasiveness and metastasis of gastric cancer. It is also possible that miR-630 serves as prognostic marker in clinical practice and even the inhibition for miR-630 using specific inhibitors may become a new therapeutic method for the treatment of gastric cancer.
